# Hairy cell leukemia presenting with Ecthyma Gangrenosum- a case report

**DOI:** 10.1186/s12879-018-3644-1

**Published:** 2019-01-25

**Authors:** R. Sluga, M. Tersmette, M. Sohne

**Affiliations:** 1 Department of Internal Medicine, St Antonius Hospital, Nieuwegein/ Utrecht, Koekoekslaan 1, 3435 CM Nieuwegein, The Netherlands; 2 Department of Medical Microbiology an Immunology, St Antonius Hospital, Nieuwegein/Utrecht, The Netherlands

**Keywords:** Ecthyma gangrenosum, Hairy-cell leukemia

## Abstract

**Background:**

Ecthyma gangrenosum is a cutaneous infectious usually associated with P. aeruginosa. It usually develops In patients with an underlying immunodeficiency.

**Case presentation:**

A 50-year old mentally disabled white male with a history of epilepsy presented with fever and a painless red macule on his right arm which rapidly progressed to a painful ulcer. Blood and lesion cultures revealed P.aeruginosa, confirming our clinical diagnosis of ecthyma gangrenosum. Subsequently an underlying immune deficit was found, namely patient was diagnosed with hairy-cell leukemia. Despite adequate antibiotics no infection control could be achieved. After treating the underlying immune deficit as well, the infection and hairy-cell leukemia resolved completely.

**Conclusion:**

Ecthyma gangrenosum is an important cutaneous infection to recognize, because it is it is typically associated with P.aeruginosa bacteremia. Recognizing this skin leasion should prompt empiric antimicrobial therapy including an agent with antipseudomonal activity. Furthermore, just like in our case, the presence of ecthyma gangrenosum can signal the presence of an occult immune deficit, warranting further investigation*.*

## Background

Ecthyma gangrenosum is an uncommon cutaneous infectious classically associated with *Pseudomonas aeruginosa* bacteremia in critically ill and immunocompromised patients. [1]

The well-recognized leasion of ecthyma gangrenosum results from perivascular bacterial invasion of the media and adventitia of arteries and veins with seconday ischemic necrosis. [2]

## Case presentation

**Fig. 1:**
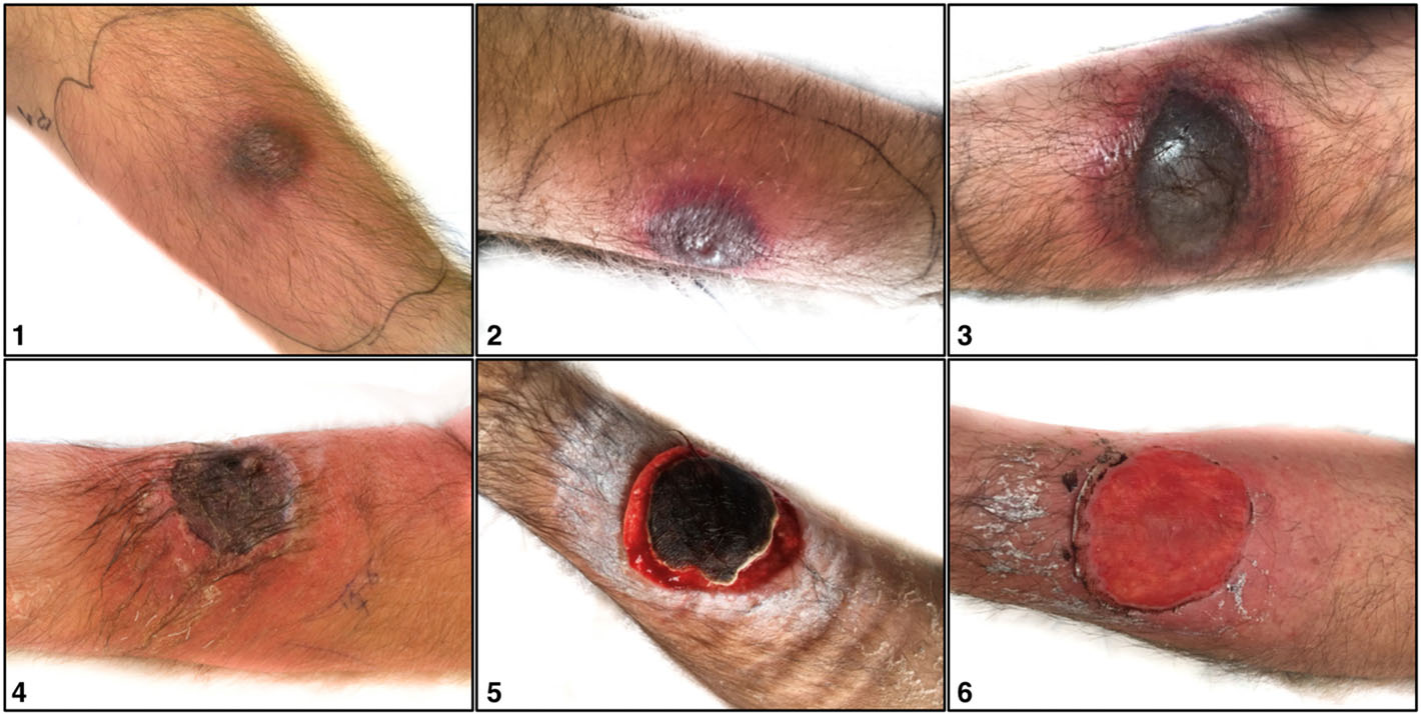
1; macule on the right anterior forearm at presentation. 2; Day 2 after presentation, macule evolving into papule. 3; Day 3 after presentation, formation of haemorrhagic bulla. 4; Day 5 after presentation, formation of necrotic ulcer. 5; Day 20 after presentation. 6; Day 30 after presentation

A 50- year old mentally disabled white male with a history of epilepsy was admitted to our hospital with fever and a painless red macule on his right anterior forearm (2x2cm) (Fig. 1) The macule had first appeared 2 days prior to presentation followed by fever since one day. Physical examination was otherwise normal. Laboratory tests showed pancytopenia (Hb 7.0 g/dl, leukocytes 1,5/mm3 with an absolute neutrophil count of 0.09/mm^3^ and thrombocytes 31/mm^3^) and elevated CRP (60 mg/l). Patient was admitted and treated empirically for erysipelas with flucloxacillin. Within 4 days the arm lesion evolved from a painless red macule into a papule, haemorrhagic bullae and ultimately into a painful ulcer suggestive of ecthyma gangrenosum (Fig. 1.). Blood and lesion cultures revealed *Pseudomonas Aeruginosa* (wild type), confirming the diagnosis. The initial empirical treatment was switched to ceftazidime.

**Fig. 2:**
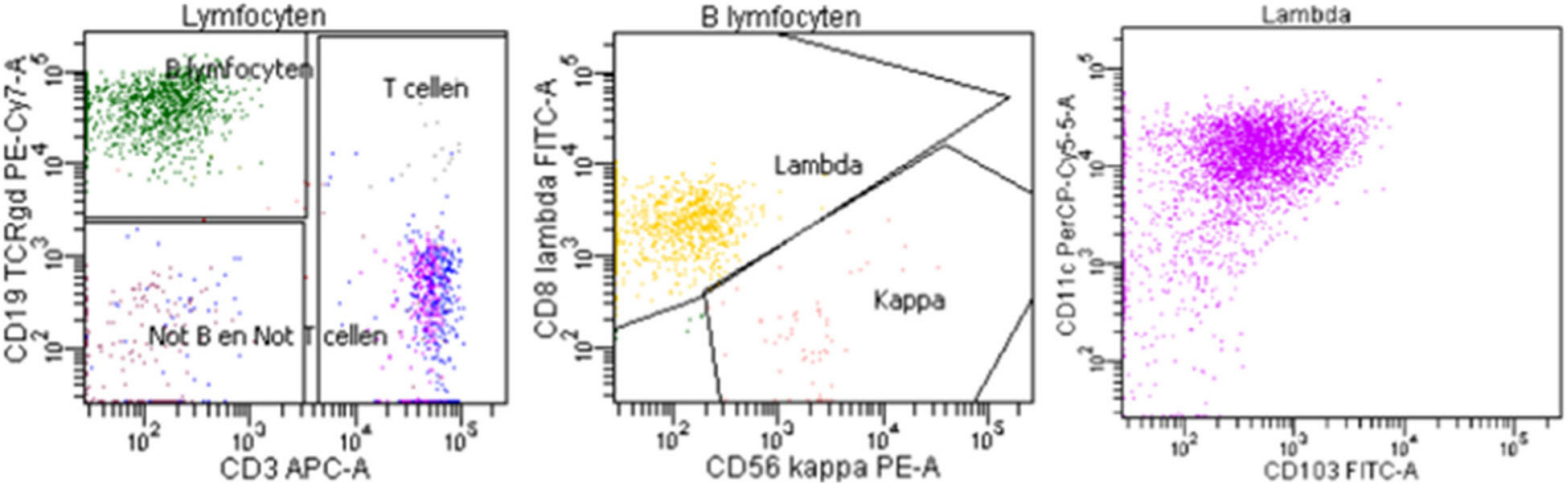
Flow cytometric dot plots show existence of abnormal cell with expression of CD103

Microscopical examination of a peripheral-blood smear revealed abnormal lymphocytes (lambda positive, monocolonal B-cell population, 4% of peripheral blood leukocytes) and immunophenotyping using the immunofluorescence with flow cytometry was positive for CD45, CD19, CD20, CD22, CD79b, CD200, CD10, CD11c,CD103, CD305 and CD25 (Fig. 2), and a diagnosis of hairy cell leukemia (HCL) was made. BRAF mutation analysis was not performed.

Despite adequate antibiotic treatment our patient continued to have high fever and elevated CRP. Clindamycin and single dose of gentamicin were empirically added to ceftazidim, but no clinical improvement ensued. We decided to start treatment of hairy cell leukaemia with cladribine (0.12 mg/kg during 5 days). After initiation of treatment, the ectyhma gangrenosum resolved completely within 3 months and the patient achieved a complete remission of HCL.

## Discussion

**Fig. 3:**
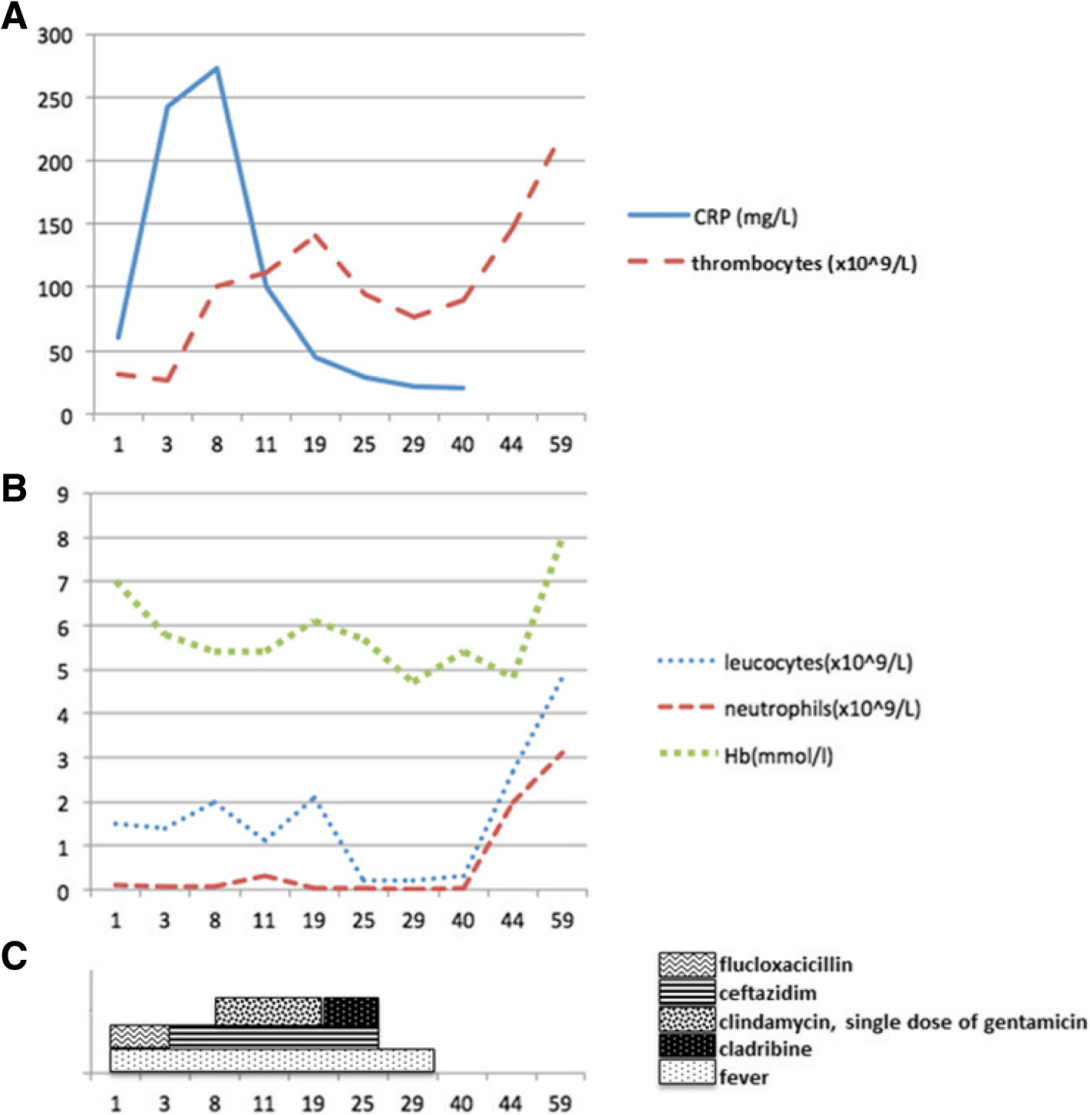
**a**; Time course (represented in days) of C-reactive protein (CRP) and thrombocytes resolution, **b**; Time course (represented in days) of leukocytes, neutrophils and hemoglobine (Hb) resolution, **c**; Time course (represented in days) of antibiotics given and fever

Ecthyma gangrenosum (EG) is a rare cutaneous ulcerative lesion mostly seen in immunocompromised patients. As seen in our patient, it characteristically starts as a painless red macule (Fig. 1, 1) that rapidly becomes pustular with surrounding erythema (Fig. 1, 2) and later haemorrhagic bullae (Fig. 1, 3). The pathogenesis of the clinical manifestation is mainly related to the invasion of the vessel walls mediated by the toxin. The subsequent ischemic necrosis results in necrotic ulcers with a black/grey eschar surrounded by an erythematous halo (Fig. 1, 4, 1.5). [3, 4]

EG is typically caused by *Pseudomonas aeruginosa*, although EG-like lesions have been observed in patients with other bacterial, viral and fungal infections. [5] The diagnosis is generally made based on the characteristic clinical appearance of the lesions along with a positive blood or wound cultures.

Ecthyma gangrenosum can signal previously undetected immunodeficiency. After presentation with ecthyma gangrenosum our patient was diagnosed with hairy-cell leukemia. HCL is an uncommon chronic B cell lymphoproliferative disorder accounting for approximately 2% of lymphoid leukemias. [6] Many patients have symptoms related to splenomegaly or cytopenias inducing fatigue, infection and/or haemorrhagic findings. Approximately one-quarter are asymptomatic, mostly presenting because of the unexpected finding of pancytopenia during routine evaluation. Patients only require treatment when they have significant cytopenia or less severe cytopenias that are symptomatic (eg., repeated infections, bleeding). Hairy cell leukemia is treated with purine analogs (ie,cladribine or pentostatin). Because these drugs are myelosupressive, it is challenging to start treatment in patients with an active infectious. [7] Since our attempt to control infection failed, we had no other option but to start treatment with the purine nucleoside analog. Treatment of the underlying disease in combination with antibiotics led to complete resolution of ecthyma gangrenosum and complete remission of HCL.

## Conclusion

In conclusion, ecthyma gangrenosum is an important cutaneous infection to recognize, because it is it is typically associated with P.aeruginosa bacteremia. Recognizing this skin leasion should prompt empiric antimicrobial therapy including an agent with antipseudomonal activity. Furthermore, just like in our case, the presence of ecthyma gangrenosum can signal the presence of an occult immune deficit, warranting further investigation.

## Abbreviations

EG: Ecthyma gangrenosum
HCL: Hairy-cell leukemia
